# Machine Learning-Based VO_2_ Estimation Using a Wearable Multiwavelength Photoplethysmography Device

**DOI:** 10.3390/bios15040208

**Published:** 2025-03-24

**Authors:** Chin-To Hsiao, Carl Tong, Gerard L. Coté

**Affiliations:** 1 Department of Biomedical Engineering, Texas A&M University, College Station, TX 77843, USA; gcote@tamu.edu; 2 School of Medicine, Texas A&M University, Bryan, TX 77807, USA; ctong@tamu.edu; 3 Center for Remote Health Technologies and Systems, Texas A&M Engineering Experiment Station, College Station, TX 77845, USA; 4 Department of Electrical and Computer Engineering, Texas A&M University, College Station, TX 77843, USA

**Keywords:** PPG, wearable sensor, oxygen consumption (VO_2_), machine learning

## Abstract

The rate of oxygen consumption, which is measured as the volume of oxygen consumed per mass per minute (VO_2_) mL/kg/min, is a critical metric for evaluating cardiovascular health, metabolic status, and respiratory function. Specifically, VO_2_ is a powerful prognostic predictor of survival in patients with heart failure (HF) because it provides an indirect assessment of a patient’s ability to increase cardiac output (CO). In addition, VO_2_ measurements, particularly VO_2_ max, are significant because they provide a reliable indicator of your cardiovascular fitness and aerobic endurance. However, traditional VO_2_ assessment requires bulky, breath-by-breath gas analysis systems, limiting frequent and continuous monitoring to specialized settings. This study presents a novel wrist-worn multiwavelength photoplethysmography (PPG) device and machine learning algorithm designed to estimate VO_2_ continuously. Unlike conventional wearables that rely on static formulas for VO_2_ max estimation, our algorithm leverages the data from the PPG wearable and uses the Beer–Lambert Law with inputs from five wavelengths (670 nm, 770 nm, 810 nm, 850 nm, and 950 nm), incorporating the isosbestic point at 810 nm to differentiate oxy- and deoxy-hemoglobin. A validation study was conducted with eight subjects using a modified Bruce protocol, comparing the PPG-based estimates to the gold-standard Parvo Medics gas analysis system. The results demonstrated a mean absolute error of 1.66 mL/kg/min and an R^2^ of 0.94. By providing precise, individualized VO_2_ estimates using direct tissue oxygenation data, this wearable solution offers significant clinical and practical advantages over traditional methods, making continuous and accurate cardiovascular assessment readily available beyond clinical environments.

## 1. Introduction

The rate of oxygen consumption (VO_2_) is a critical parameter in assessing cardiovascular, respiratory, and metabolic health [1,2,3,4]. It reflects the ability of the cardiopulmonary system to deliver oxygen and the body’s metabolic needs. It is widely used in evaluating exercise performance, monitoring chronic conditions, and diagnosing diseases such as heart failure and pulmonary disorders [5,6]. However, conventional methods for measuring VO_2_ require bulky and often expensive gas analysis systems, which limit their accessibility and practicality for routine use. This constraint has motivated researchers to explore alternative, non-invasive techniques for continuous VO_2_ monitoring.

**Figure 1:**
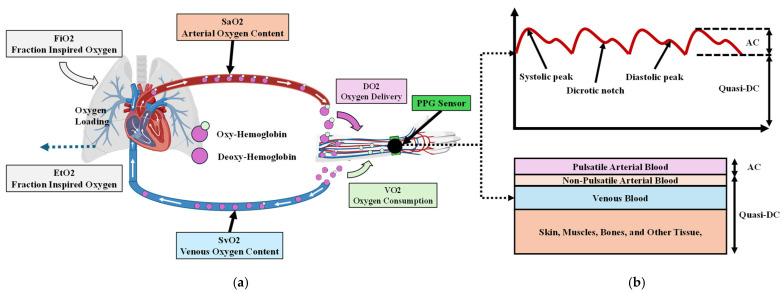
(**a**) Simplified model of a closed-loop cardiovascular system with a heart, lungs, and a left arm. (Created with BioRender.com (accessed on 14 December 2024)) (**b**) PPG waveforms with the features (systolic peak, dicrotic notch, and diastolic peak) and the AC and quasi-DC component.

The cardiovascular system consists of three main components: the heart, blood vessels, and blood [7]. As depicted in Figure 1a, the system operates as a closed-loop model. Re-oxygenated blood enters the left atrium from the lungs and flows into the left ventricle, from where it is pumped through the aorta to deliver oxygen via major arteries to various parts of the body. For example, blood flows through the radial artery to the capillaries in the left arm, then returns via the radial vein to the subclavian vein, then to the superior cava, ultimately reaching the right atrium and continuing to the lungs via the pulmonary artery for reoxygenation.

During this process, the fractional inspired oxygen (FiO_2_) represents the oxygen concentration in inhaled air, typically around 21% in room air [8], while end-tidal oxygen (EtO_2_) reflects the oxygen concentration in exhaled air. The arterial oxygen saturation (SaO_2_) indicates the oxygen content in arterial blood, and the venous oxygen saturation (SvO_2_) measures the oxygen content in venous blood. VO_2_ represents the difference in oxygen content before and after tissue delivery, providing a critical metric for cardiopulmonary delivery and metabolic activity [9].

Photoplethysmography (PPG) is a non-invasive optical method that measures blood volume changes within tissue, offering insights into cardiovascular dynamics. It is commonly used to measure physiological parameters such as heart rate, oxygen saturation (SpO_2_), and blood pressure [10,11,12]. As shown in Figure 1b, the PPG signal consists of an alternating current (AC) component, reflecting pulsatile changes caused by cardiac cycles that capture dynamic features such as heart rate and arterial compliance [13]. It also includes a quasi-direct current (DC) component, which represents baseline tissue and blood volume changes and holds untapped potential for analyzing slower occurring changes, such as cardiac output (low cardiac output will depress O_2_ content of this DC component). Most studies focus on the AC component for extracting cardiovascular metrics [14,15,16], utilizing its amplitude and periodicity to estimate metrics such as heart rate, blood pressure, stress levels, and beat-to-beat intervals [17,18,19,20,21,22]. However, the DC signal reflects the venous blood flow, diastolic portion of the arterial flow, tissue content, and tissue optical properties, providing information about overall blood perfusion. Thus, we focused on the quasi-DC portion of the PPG waveform to explore its relationship with VO_2_ in the forearm’s tissue, blood, and muscle, as shown in Figure 1b.

The traditional assessment of oxygen consumption (VO_2_) primarily relies on indirect calorimetry using metabolic carts, which measure the volume and concentration of inhaled and exhaled gases (primarily oxygen and carbon dioxide) breath-by-breath during physical exertion [23]. These systems are considered the gold standard because of high accuracy but have significant limitations. Metabolic carts are typically bulky, costly, and require controlled laboratory settings, trained personnel, and calibration before each measurement, which restricts their utility for continuous, real-world monitoring [24,25]. Furthermore, invasive approaches, such as the direct Fick method, involve arterial and venous catheterization to measure arterial-venous oxygen differences, presenting risks associated with invasive procedures, patient discomfort, and potential complications, thereby limiting their routine clinical applicability [26]. These drawbacks emphasize the need for alternative, non-invasive methods capable of continuous, real-time VO_2_ monitoring in various settings, motivating the development of innovative wearable technologies such as the multiwavelength PPG device proposed in this study.

In this study, we developed and tested a novel approach to estimate VO_2_ using a wrist-worn PPG device equipped with a multi-wavelength sensor [27]. By leveraging the Beer–Lambert law and analyzing the DC component of PPG signals using a novel machine learning model, we aimed to uncover insights into the static optical properties of the tissue, which correlate with metabolic activity and oxygen utilization. This expands the utility of PPG beyond conventional AC-based measurements to provide a deeper understanding of tissue-level oxygen dynamics. This will overcome the limitations of current measurement systems by addressing the need for compact, accessible, and continuous VO_2_ monitoring systems and will contribute to the growing field of wearable health technologies, with implications for personalized healthcare and chronic disease management.

## 2. Materials and Methods

Our wearable device includes a multi-spectral photoplethysmography (PPG) sensor [27] to measure VO_2_ non-invasively. By leveraging five wavelengths of light (670 nm, 770 nm, 810 nm, 850 nm, and 950 nm), including an isosbestic point at 810 nm, to enhance sensitivity to hemoglobin dynamics, this advanced sensor captures detailed optical signals on the palmar side of the arm, and uses the Beer–Lambert Law to enable differentiation between oxy-hemoglobin and deoxy-hemoglobin.

### 2.1. Beer–Lambert Law

The Beer–Lambert law is a fundamental principle in optical absorption spectroscopy, relating the attenuation of light to the properties of the material through which the light passes [28]. Mathematically, it expresses the relationship between the molar extinction coefficient (ε), the concentration (C), and the path length (l) as follows [28]:

(1) $$I=I_{0}\times e^{-\varepsilon cl}$$

**Figure 2:**
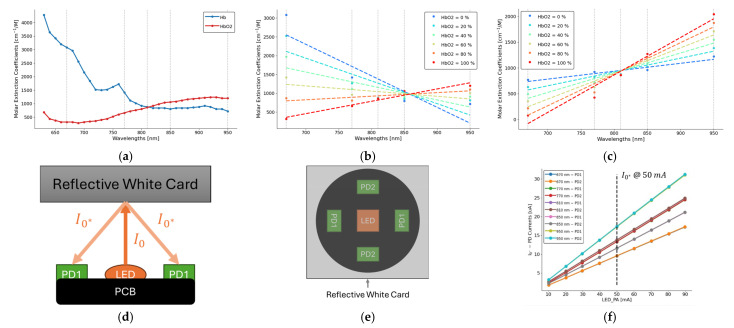
(**a**) Molar extinction coefficient of oxy-hemoglobin (HbO_2_) and deoxy-hemoglobin (Hb) from 600 nm to 950 nm. (**b**) Interpolation of molar extinction coefficient from Hb (0%) and HbO_2_ (100%) at 5 wavelengths (670 nm, 770 nm, 810 nm, 850 nm, and 950 nm) at a step of 20% increase. The dashed line represents the regression at each oxygen level. (**c**) Molar extinction coefficient multiplying a scaling factor α(λ) to move the intercept to 810 nm. (**d**) Illustration of a reflective white card setup to estimate the $I_{0}^{*}$ (cross-sectional view). (**e**) Illustration of a reflective white card setup to estimate the $I_{0}^{*}$ (top view). (**f**) The received photodiode currents of $I_{0}^{*}$ in terms of LED pulse amplitude (PA). The black dashed line represents the corresponding $I_{0}^{*}$ at LED PA of 50 [mA].

In this equation, I represents the transmitted light and $I_{0}$ represents the incident light. We applied the Beer–Lambert law using the extinction coefficients of oxy-hemoglobin (HbO_2_) and deoxy-hemoglobin (Hb) [29], at five distinct wavelengths (670 nm, 770 nm, 810 nm, 850 nm, and 950 nm), as shown in Figure 2a. The following describes the relationship between light attenuation and the concentrations of HbO_2_ and Hb [28]:

(2) $$I\left(\lambda \right)=I_{0}(\lambda )\times e^{-{[\varepsilon }_{Hb}\left(\lambda \right)C_{Hb}+\varepsilon_{HbO2}\left(\lambda \right)C_{HbO2}]l(\lambda )}$$

Figure 2b illustrates the interpolation of four points (20%, 40%, 60%, 80%) between 0% oxygenation (Hb) and 100% oxygenation (HbO_2_) in the blood. By performing a linear regression on these six oxygenation levels for the extinction coefficients across the five wavelengths, we observe changes in the slope of the fitted lines as oxygenation decreases. This observation underpins our approach to correlating the slope of these lines with oxygen consumption (VO_2_).

Figure 2c highlights the isosbestic point of Hb and HbO_2_ extinction coefficients at 810 nm. To standardize the analysis, we scale the extinction coefficients to align their intercept points by multiplying by a factor, α(λ), as follows [28]:

(3) $$I\left(\lambda \right)=I_{0}(\lambda )\times e^{-{[\varepsilon }_{Hb}\left(\lambda \right)C_{Hb}+\varepsilon_{HbO2}\left(\lambda \right)C_{HbO2}]l(\lambda )\times \alpha (\lambda )}$$

The absorbance ($A_{Hb}(\lambda )$) of the hemoglobin (Hb and HbO_2_) is defined in Equation (4). This adjustment ensures consistency across the wavelengths and enhances the reliability of the calculated correlations with VO_2_.

(4) $$A_{\mathrm{Hb}}(\lambda )=-\ln(\frac{I\left(\lambda \right)}{I_{0}\left(\lambda \right)})={[\varepsilon }_{Hb}\left(\lambda \right)C_{Hb}+\varepsilon_{HbO2}\left(\lambda \right)C_{HbO2}]l\left(\lambda \right))\times \alpha (\lambda )$$

To measure the incident light $I_{0}$, we developed a simple setup with a reflective white card, as shown in Figure 2d. The reflective light was transmitted to all 4 photodiodes as shown in Figure 2e. In this study, we combined photodiodes input horizontally (PD1) and vertically (PD2) into one signal. During the measurement process, we adjusted the LED pulse amplitude/intensity from 10 mA to 90 mA. The response in photodiode currents $I_{0^{*}}$ is shown in Figure 2f. Each wavelength has 2 response curves from PD1 and PD2. In this study, we used 50 mA for all wavelengths during the entire data collection process. Assuming $I_{0}\approx I_{0}^{*}$ and $l\left(\lambda \right)$ is linear [30], the Beer–Lambert law provides a robust framework for leveraging multi-wavelength optical data to quantify oxygenation dynamics and to estimate oxygen consumption.

### 2.2. PPG Sensor System

**Figure 3:**
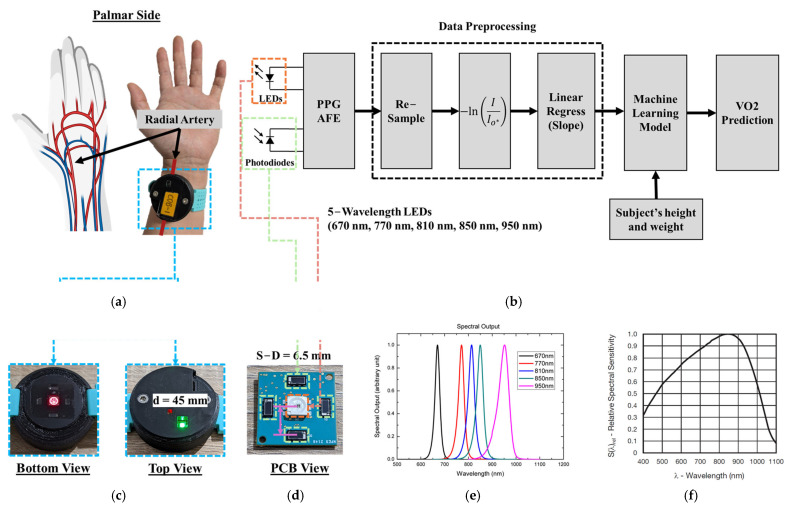
(**a**) An illustration of sensor location and demonstration of the target arteries and veins. (created with BioRender.com (accessed on 14 December 2024)) (**b**) Signal processing chain from LEDs and PDs to VO_2_ prediction (machine learning algorithm output) (**c**) Bottom and side view of the 3D printed mechanical/industrial design of the wearable PPG sensor. (**d**) PCB view of showing the source (LEDs) to detector (PDs) distance is 6.5 mm for all PDs (**e**) The LEDs spectral output of 5 wavelengths (adapted from the datasheet [31]) (**f**) The relative spectral sensitivity of the photodiode (adapted from the datasheet [32]).

The PPG sensor system was designed as a wrist-worn device targeting the radial artery, as described in a prior study [27]. The palmar side of the wrist was selected as the sensing location instead of the dorsal side due to the presence of major arteries and veins in the palmar region. Specifically, this study targeted the region of the radial artery and radial vein, as illustrated in Figure 3a.

Figure 3b depicts the signal processing chain, starting with the acquisition of PPG waveforms via the analog front-end (AFE). The initial sampling rate was set to 100 Hz but was down sampled to 0.1 Hz to focus on the quasi-DC components of the PPG signal, which changed more gradually compared to heart rate fluctuations. Using the photodiode current values $I_{0^{*}}$ obtained from the lookup table in Figure 2f, the hemoglobin absorbance $A_{Hb}\left(\lambda \right)$ was calculated. Linear regression of $A_{Hb}\left(\lambda \right)$ was performed to compute the slope every 10 s for photodiodes PD1 and PD2, yielding slope values $m_{1}$ and $m_{2}$, respectively.

Consequently, these slope values ($m_{1}$ and $m_{2}$), combined with anthropometric measurements (height and weight) of the subjects, were used to train a random forest machine learning model. The model was developed to predict VO_2_ values using the metabolic measurement system (TrueOne 2400, Parvo Medics, Salt Lake City, UT, USA) as the reference.

The mechanical design of the device features a diameter of 45 mm, as shown in Figure 3c. The printed circuit board (PCB) incorporates a source-to-detector distance of 6.5 mm, as illustrated in Figure 3d. A multichip LED (MTMD6788594SMT6, Marktech Optoelectronics, Latham, NY, USA) was selected for this study. This LED integrates five wavelengths (670, 770, 810, 850, and 950 nm) within a single IC package, with its spectral output shown in Figure 3e [31]. The photodiode (VEMD8080, Vishay, Malvern, PA, USA) used in this device has its sensitivity profile displayed in Figure 3f [32].

### 2.3. Human Subject Study Protocol

**Figure 4:**
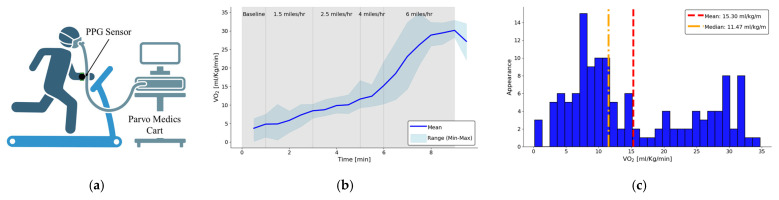
(**a**) An illustration of the human subject study protocol setup with the proposed PPG sensor and reference device Parvo Medics TrueOne 2400 system (Created with BioRender.com (accessed on 14 December 2024)) (**b**) The average variation and range in VO_2_ [ml/kg/m] while running at 4 different speed (1.5, 2.5, 4, and 6 mile/h) from 8 subjects (**c**) The distribution of the VO_2_ [ml/kg/m] collected from the reference deice averaging at 15.3 mL/kg/m.

To validate the algorithm, a human subject study was conducted using a modified Bruce protocol [33], the protocol setup is shown in Figure 4a. Informed written consent was obtained, and the study was conducted under IRB2021-0962F, approved by the Texas A&M University Institutional Review Board (IRB). This exercise protocol was specifically designed to include low-intensity activities that naturally elevate heart rate and cardiac output (CO) without overexertion. Increasing CO will cause an increase in VO_2_. A total of 11 subject’s data were collected and 3 subject’s data were excluded due to the incomplete PPG data that was corrupted during the data saving process.

The participants performed a series of walking and jogging intervals on a treadmill with a flat incline, ensuring accessibility for individuals with varying fitness levels. The protocol began with warm-up periods at progressively increasing walking speeds, followed by a short jogging session to further stimulate cardiovascular activity, the exercise protocol is shown in Table 1.

Measurements were taken intermittently throughout the exercise, including data from the wrist-worn PPG device and a breath analyzer for oxygen consumption (VO_2_). The VO_2_ [mL/kg/min] trend during the exercise period from the 8 subjects is shown in Figure 4b and the distribution of all the VO_2_ [mL/kg/min] is shown in Figure 4c. Additionally, post-exercise echocardiography assessments were conducted to evaluate CO (CO = stroke volume (SV) X heart rate (HR)) changes and their correlation with the derived VO_2_ measurements.

## 3. Results

**Figure 5:**
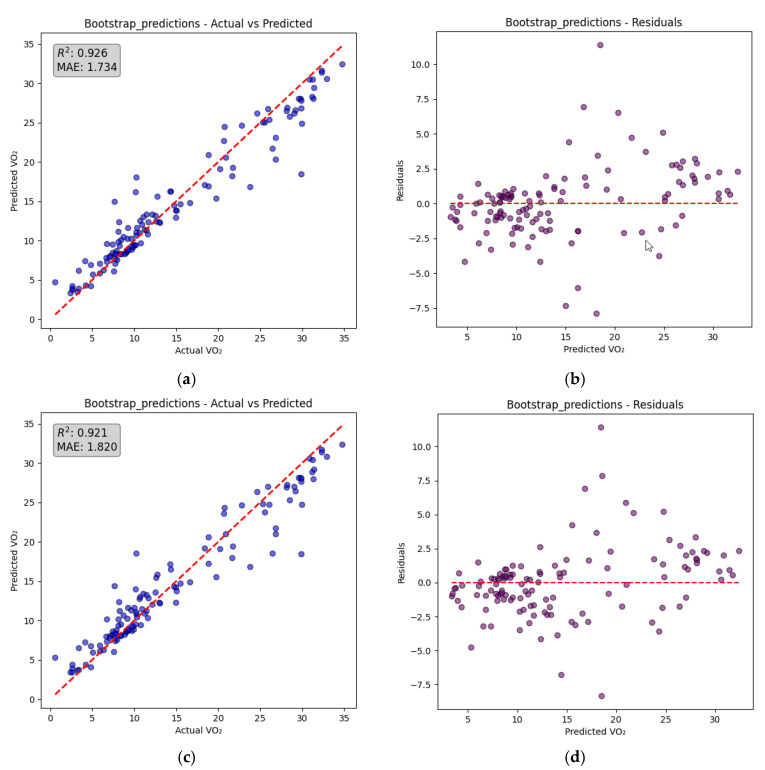
(**a**) A prediction curve (red dashed line) of VO_2_ [mL/kg/min] using five wavelengths as an input of machine learning model. (**b**) A residual plot that shows the mean error (red dashed line) and distribution of the error using five wavelengths as an input of machine learning model. (**c**) A prediction curve (red dashed line) of VO_2_ [mL/kg/min] using three wavelengths as an input of machine learning model. (**d**) A residual plot that shows the mean error (red dashed line) and distribution of the error using three wavelengths as an input of machine learning model.

Figure 5a,b display the prediction curve and residuals plot for the random forest machine learning model using bootstrap aggregation (bagging) with five wavelengths. Bootstrap aggregation is a technique where multiple decision trees are trained on different subsets of the data, sampled with replacement, to reduce overfitting and improve prediction accuracy. The final prediction is obtained by averaging the outputs of all the trees in the ensemble, making the model robust to variability in the data.

It should be noted that we explored several regression-based algorithms, including linear regression, in the initial stages of model development. However, we observed that the relationships between our selected features and VO_2_ were inherently non-linear and complex, making linear regression methods inadequate without extensive transformations, which might risk introducing bias or reducing interpretability. Consequently, we selected the Random Forest algorithm due to its robustness in capturing complex, non-linear relationships, resistance to overfitting, and minimal need for feature transformation. Additionally, we utilized bootstrap predictions to further enhance model reliability and assess its generalization capability. While deep learning methods were considered, they typically require significantly larger datasets for optimal performance. Given the limited sample size in our study, Random Forest provided an ideal balance between complexity, interpretability, and accuracy.

Using this approach, the model achieved a mean absolute error (MAE) of 1.73 [mL/kg/min] and an R-squared value of 0.93. In comparison, as shown in Figure 5c,d, using only three wavelengths resulted in an MAE of 1.82 [ml/kg/m] and an R-squared value of 0.92. This demonstrates the potential to achieve similar accuracy with a reduction in the number of LEDs used in the algorithm to simplify the design.

In Figure 5, both the prediction accuracy (actual vs. predicted VO_2_) and residual analysis for the 5-wavelength and 3-wavelength PPG models are shown. Examining the residual plots as shown in Figure 5b,d, it is evident that residuals are randomly distributed around zero with no clear or systematic pattern. This indicates that the prediction errors are unbiased and consistent across the entire range of predicted VO_2_ values, demonstrating that the model performance does not significantly degrade at low, moderate, or high levels of VO_2_. A slight increase in variance can be observed at higher VO_2_ values (above ~25 mL/kg/min), which is expected due to physiological variability at higher intensities. However, this variability remains minimal, further reinforcing the robustness of our multiwavelength PPG-based VO_2_ estimation approach.

## 4. Discussion

The findings of this study highlight both the benefits and limitations of using the quasi-DC component of PPG waveforms for estimating rate of oxygen consumption (VO_2_).

### 4.1. Comparison with Existing Wearable VO_2__max Estimation Methods

Traditional wearable fitness devices estimate VO_2__max using static formulas that primarily rely on heart rate (HR) data and user demographics. For instance, the Polar A300™ estimates VO_2__max from resting cardiac variability [34], while devices like the Apple Watch and Fitbit utilize HR and activity data during exercises to predict VO_2__max [35,36,37]. These methods, while convenient, often lack the precision of direct measurements due to their reliance on generalized algorithms that do not account for individual physiological variations.

Recent studies have sought to enhance the accuracy of VO_2__max estimation by integrating additional data sources. For example, Spathis et al. [38] developed a model predicting cardiorespiratory fitness using wearable sensor data, including HR and movement metrics, collected during daily activities, achieving a strong correlation with laboratory measurements. Similarly, a study by Amelard et al. [39] employed temporal convolutional networks to predict dynamic oxygen uptake responses from wearable sensors across various exercise intensities, demonstrating the potential of advanced machine learning techniques in this domain.

In contrast to these approaches, our study introduces a novel method for continuous VO_2_ estimation by leveraging oxygenation information obtained directly from a custom-designed, multiwavelength photoplethysmography (PPG) wearable sensor. This technique allows for real-time monitoring of VO_2_ without relying solely on HR or demographic data, thereby providing a more individualized and precise assessment. Our validation study demonstrated a mean absolute error of 1.66 mL/kg/min and an R^2^ of 0.94 when compared to the gold-standard Parvo Medics gas analysis system, indicating a high level of accuracy. By directly measuring oxygenation changes through PPG signals, our approach addresses the limitations of static formula-based estimations and enhances the potential for continuous, non-invasive VO_2_ monitoring in both clinical and everyday settings. Table 2 summarizes the comparison of our method with previously published wearable-based VO_2_ estimation studies.

### 4.2. Advantages of DC Analysis

In prior research [17,18,19,20,21,22], the AC component of PPG waveforms has been extensively utilized for extracting morphological features and amplitude variations, which are strongly correlated with cardiovascular metrics such as heart rate, blood pressure, and cardiac output. However, the primary limitation of relying on the AC component lies in its high susceptibility to motion artifacts [40,41,42,43,44,45].

**Figure 6:**
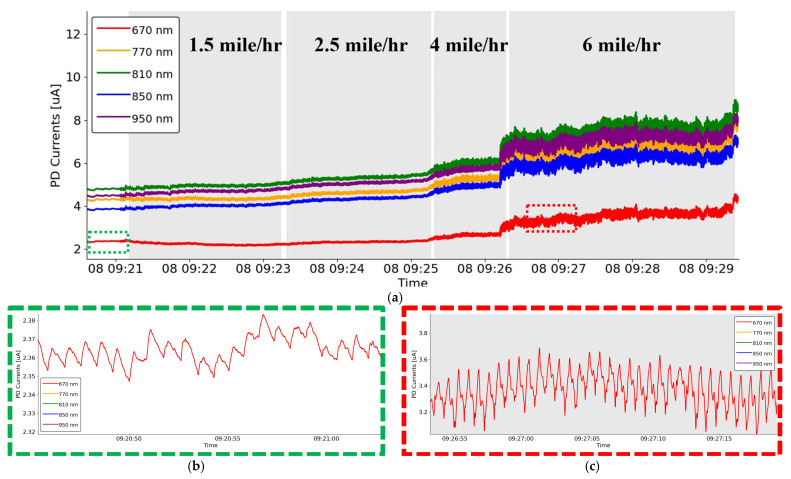
(**a**) The raw PPG waveforms of five wavelengths from stationary to running. (**b**) A zoom-in view of the PPG waveform from 660 nm during the stationary period before starting running. (**c**) A zoom-in view of the PPG waveform from 660 nm while running at the speed of 6 miles per hour.

This sensitivity is evident in Figure 6a, which displays the raw PPG waveforms from the analog front-end (AFE) for a single subject. While the zoomed-in window of the 660 nm waveform in Figure 6b illustrates the pulsatile signal produced by heartbeats during resting conditions, Figure 6c shows how motion during exercise severely disrupts the AC component, rendering it virtually impossible to discern heart rate signals or morphological features in the time domain.

In contrast, the quasi-DC component, as seen in Figure 6a, demonstrates significantly less sensitivity to motion artifacts. The gradual variations in the DC signal provide a robust metric for analysis without relying on beat-to-beat heart rate signals, offering a new avenue for extracting physiological information during dynamic activities.

Another advantage is that VO_2_ measurement provides critical medical physiology measurements that which arterial pulsation (AC) portion cannot do. VO_2_ consists of the body’s metabolic demand, the heart’s ability to vary cardiac output, and the pulmonary system’s ability to provide O_2_ exchange. In contrast, the AC portion only measures the pulmonary system’s ability to deliver O_2_ to the arterial system. Knowing VO_2_ can be used to quantify a wide span of human conditions from the severity of heart failure (low VO_2_) to sports performance (high VO_2_). Being light and portable, this system then can be used to guide outpatient treatment of heart failure patients, which has a current US prevalence of 6.7 M [46].

### 4.3. Limitations of the Study

The study’s findings are limited by the relatively homogeneous population of healthy subjects aged 21 to 31. Data from older adults or individuals with various health conditions, such as cardiovascular or respiratory diseases, were not included. Expanding the dataset to incorporate a more diverse population is essential to validate and generalize the algorithm’s applicability across different demographics and health states. Future research should aim to address these limitations by collecting data from larger, more diverse cohorts and further refining the algorithm to accommodate varying physiological conditions.

## 5. Conclusions

This study presents a novel approach to estimating oxygen consumption (VO_2_) non-invasively using a multi-wavelength photoplethysmography (PPG) wearable device and a random forest machine learning algorithm. By leveraging the Beer–Lambert law and analyzing the quasi-DC components of PPG waveforms, we demonstrated a robust methodology to overcome the limitations of conventional VO_2_ measurement systems, which rely on bulky gas analysis equipment. The results showed that the proposed system, which utilized five distinct wavelengths (670 nm, 770 nm, 810 nm, 850 nm, and 950 nm), achieved high accuracy with a mean absolute error (MAE) of 1.66 mL/kg/min and an $R^{2}$ value of 0.94 in predicting VO_2_. Unlike traditional wearable devices that rely solely on heart rate or demographic data, our method directly utilizes real-time oxygenation information, enabling individualized and precise VO_2_ monitoring. This highlights the advantage of multi-spectral sensing in capturing comprehensive hemoglobin dynamics for VO_2_ estimation. Additionally, the use of the DC component of the PPG signal provides a major advantage over the traditionally used AC component, particularly in reducing sensitivity to motion artifacts during exercise. This enables continuous monitoring of VO_2_ even under dynamic conditions, such as physical activity, where AC analysis is typically unreliable. This prediction approach has significant implications for other biomedical applications such as non-invasive cardiac output monitoring. By providing a reliable estimate of oxygen consumption (VO_2_), the system has the potential to indirectly assess cardiac output, offering a valuable tool for personalized healthcare and fitness monitoring. This innovation could lead to broader applications in clinical diagnostics, exercise physiology, and chronic disease management, advancing the capabilities of wearable health technologies. Future studies should expand validation to larger, more diverse populations to further confirm generalizability and reliability.

## Figures and Tables

**Table 1:** Human subject study protocol with walking and running on the treadmill at different speeds.

Exercise Protocol	Duration	Comment
Sitting Sedentary	0.5 min	Establish PPG baseline
Walking	2 min	1.5 miles/h
Walking	2 min	2.5 miles/h
Running	1 min	4 miles/h
Running	3 min	6 miles/h
Total	~10 min	

**Table 2:** Benchmark comparison of the current study with existing literature.

Study	Methodology	Sample Size	Advantages	Limitations
**Fitbit Charge 2 (Freeberg et al., 2019) [35]**	HR + activity data estimation	30	Convenient; Large user base	Relies on static formulas; less precise
**Apple Watch Series 7 (Caserman et al., 2024) [36]**	HR-based estimation during exercise	19	Widely accessible; User-friendly	Lower precision; Relies on demographic and HR data only
**Neural Network model (Spathis et al., 2022) [38]**	HR + accelerometer data	11,059	Large, diverse dataset; Effective in daily life settings	High computational cost; No oxygenation data
**Temporal convolutional network (Amelard et al., 2021) [39]**	Wearable sensors with advanced ML	22	Captures dynamics at varying intensities; Robust	Requires extensive data and computational power
**This study**	Multiwavelength PPG + Random Forest	8	Direct oxygenation measurement; Real-time; Non-invasive; High accuracy	Small sample; Preliminary validation

## Data Availability

The data presented in this study are available on request from the corresponding author.
